# Giant primary scrotal lipoma in a low-resource setting: challenges with diagnosis and review of literature

**DOI:** 10.1093/jscr/rjab398

**Published:** 2021-09-22

**Authors:** Anwar Sadat Seidu, Joseph Yorke, Joseph Akpaloo, Patrick Danso, Seidu Suhewie Sukenibe, Papa Kwesi Fiifi-Yankson, Kwadwo Adae-Aboagye, George Amoah, Francis Akwaw Yamoah, Denis Afful-Yorke, Samuel Nana Prempeh Agyeman-Gyebi, Samuel Gyasi Brenu, Ernest Adjei

**Affiliations:** Department of Surgery, Tamale Teaching Hospital, Tamale, Ghana; Department of Surgery, Kwame Nkrumah University of Science and Technology, Kumasi, Ghana; General Surgery Unit, Komfo Anokye Teaching Hospital, Kumasi, Ghana; Department of Surgery, Kwame Nkrumah University of Science and Technology, Kumasi, Ghana; Plastics and Reconstructive Surgery Unit, Komfo Anokye Teaching Hospital, Kumasi, Ghana; General Surgery Unit, Komfo Anokye Teaching Hospital, Kumasi, Ghana; Plastics and Reconstructive Surgery Unit, Komfo Anokye Teaching Hospital, Kumasi, Ghana; Department of Surgery, Kwame Nkrumah University of Science and Technology, Kumasi, Ghana; Plastics and Reconstructive Surgery Unit, Komfo Anokye Teaching Hospital, Kumasi, Ghana; Plastics and Reconstructive Surgery Unit, Komfo Anokye Teaching Hospital, Kumasi, Ghana; Urology Unit, Komfo Anokye Teaching Hospital, Kumasi, Ghana; General Surgery Unit, Komfo Anokye Teaching Hospital, Kumasi, Ghana; General Surgery Unit, Komfo Anokye Teaching Hospital, Kumasi, Ghana; General Surgery Unit, Komfo Anokye Teaching Hospital, Kumasi, Ghana; General Surgery Unit, Komfo Anokye Teaching Hospital, Kumasi, Ghana; Department of Pathology, Komfo Anokye Teaching Hospital, Kumasi, Ghana

## Abstract

Lipoma is the commonest benign mesenchymal tumor composed of matured adipocytes. A literature search revealed few reported cases of giant scrotal lipoma. This article aims to report a giant scrotal lipoma weighing 1100 g and illustrate our challenges with the diagnostic process in a low-resource setting. A 28-year-old male presented with a huge right scrotal mass. Examination revealed the mass had no cough impulse. It was firm, non-tender and lobulated, with definite edges. Scrotal sonography was suspicious of lipoma. Intraoperatively, there was an encapsulated scrotal wall mass and an incidental inguinoscrotal hernia, content being the omentum. The scrotal mass was excised, hernia sac was ligated, and excised and the posterior wall was repaired. Histology confirmed the scrotal mass as a lipoma. Primary scrotal lipomas are rare but should be considered in the differential diagnosis of unusual scrotal masses. Ultrasonography is a useful diagnostic tool in resource-limited settings.

## INTRODUCTION

Lipoma is the commonest benign mesenchymal tumor composed of matured fat cells [1, 2]. It may arise from various anatomic locations, the scrotum being the least [1, 2]. Lipoma of the scrotum may originate from different anatomic structures of the scrotum [3]. A giant scrotal lipoma is a rare clinical occurrence [4, 5]. Unusual scrotal masses have atypical clinical presentation that poses diagnostic challenges to the clinician [6, 7]. Radiological imaging may aid in preoperative diagnosis and localization of scrotal lesions [8]. The best treatment modality for lipoma of the scrotum is surgical resection [3, 6]. A literature search revealed few reported cases of giant scrotal lipoma weighing 480–700 g [9, 10, 12].

This article aims to report a 1100-g giant scrotal lipoma in a young male and review the literature. We also illustrate our challenges with the diagnostic process in a resource-limited setting.

## CASE SUMMARY

**Figure 1:**
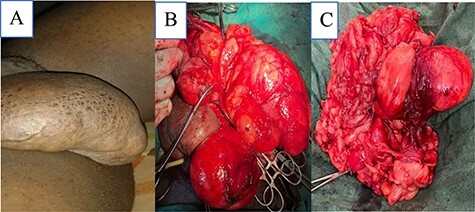
(**A**) Huge right scrotal mass, (**B**) intraoperative image of scrotal mass and (**C**) excised scrotal mass weighing 1100 g.

**Figure 2:**
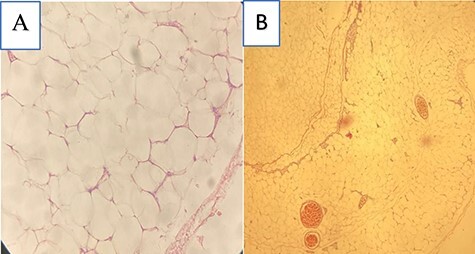
Microscopic section of scrotal mass (**A**) ×40 lobules of matured lipocytes with a fibrous capsule and (**B**) ×40 matured lipocytes with few blood vessels.

A 28-year-old male presented with a gradually increasing right inguinoscrotal swelling over 3 years. There were no other associated symptoms. On clinical examination, he was afebrile, not pale and well hydrated. Systemic examination was normal. The abdomen was flat with no visible and palpable cough impulse. There was a huge right hemiscrotal mass which measured 25 × 12 × 8 cm, irreducible, firm, non-tender and lobulated with definite edges as shown in Fig. 1A. It did not transilluminate to light. The right spermatic cord was not palpable. There were no palpable inguinal lymph nodes. Scrotal ultrasonography (USG) reported a large extra-testicular homogenous echotexture of fat lobules arising from the scrotal wall suggestive of a lipoma. Both testes were normal. Color doppler showed normal flow. The patient was counseled for an excision biopsy under spinal anesthesia. Intraoperatively, a longitudinal incision on the right hemiscrotum was made and deepened through the spermatic fascia and cremaster to expose the scrotal mass. A giant multilobulated fatty scrotal wall mass was noted extending superiorly into the inguinal canal as shown in Fig. 1B. There was also a hernia sac identified within the scrotum. A transverse inguinal incision was made and dissected to expose the external oblique aponeurosis. The aponeurosis was opened along its fibers to enter the inguinal canal. The spermatic cord was delivered into the wound and the indirect sac was identified and dissected free from the cord. The sac was opened revealing omental tissue, which was reduced. The sac was ligated with vicryl 0 and excised. The weak posterior wall was repaired with nylon 2 using the Halsted technique. Wounds were closed in layers with vicryl 2/0. Skin closed with vicryl 2/0. The patient was discharged on Day 2. Histology of the excised mass was reported as weighed 1100 g and measured 21 × 7 × 9 cm. It appeared yellowish in color and had a fibrous capsule. Microscopic sections showed matured adipocytes arranged in lobules separated by thin fibrous septae as shown in Fig. 2A and B. The histopathological diagnosis was a scrotal lipoma.

## DISCUSSION

Common scrotal masses like hydrocele, varicocele and inguinoscrotal hernia are often managed successfully by general practitioners in resource-limited settings. Clinical history and examination are often enough to make diagnose of common scrotal masses. Atypical scrotal masses are difficult to diagnose and are often referred to the urologist for expert management. A giant primary scrotal lipoma is a rare clinical occurrence that poses preoperative diagnostic challenges to the clinician [4, 5]. But with appropriate clinical and radiological tools, successful diagnosis is attainable [5]. In this report, we presented a 28-year-old male with a long-standing right inguinoscrotal mass with atypical examination findings. Uncommon scrotal masses are difficult to diagnose by clinical evaluation alone [6]. The majority of extra-testicular masses are benign [13]. An adenomatoid tumor is the commonest benign extra-testicular tumor with an incidence of 30% [8]. Lipoma may arise from three main tissues in the scrotum; adipose tissue of the spermatic cord, spermatic cord and the scrotal wall [3]. In this case, the lipoma originated from the scrotal wall. Occasionally, some lipomas may herniate into the scrotum from retroperitoneal structures [14]. An extensive literature search revealed few cases of giant scrotal lipoma weighing between 480 and 700 g [9, 10, 12]. In this report, the scrotal lipoma weighed 1100 g which in our view is probably the largest in literature.

The use of radiological imaging provides invaluable information that may aid in preoperative diagnosis and surgical planning [8, 13]. USG is the cheapest and readily available imaging modality for scrotal lesions [8, 13]. In low-resource settings, this may be the only imaging tool available to clinicians. It is worth noting that USG is operator-dependent and is only an excellent tool in experienced hands. In this report, scrotal USG reported a large extra-testicular homogenous echotexture of fat lobules confirming a scrotal lipoma but failing to identify an ipsilateral inguinoscrotal hernia. Sonographic features of scrotal lipoma are a homogenous echogenic pattern compared to the surrounding structures [8, 14]. USG is not routinely used for diagnosing hernia. But when the clinical findings are equivocal, it may aid in diagnosis [14]. USG may show a hernia sac containing air or fluid-filled loops of bowels or a complex echogenic fatty mass if the omentum is involved [8]. The sonographer in this report missed an incidental ipsilateral inguinoscrotal hernia. Differentiating overlapping inguinoscrotal hernia containing omentum and a huge scrotal lipoma on USG may be difficult, especially so by an inexperienced sonographer. The missed diagnosis may have informed the initial scrotal surgical approach to the pathology. Perhaps, a computed tomography (CT) scan or magnetic resonance imaging scan may have avoided the missed preoperative diagnosis in this case [5].

Liposarcoma of the spermatic cord is a differential diagnosis of clinical importance in this case. It accounts for 3–7% of all extra-testicular tumors [15]. It grows slowly and spread via hematogenous and lymphatic channels. There is no standardized management for these liposarcomas due to paucity of clinical data. The current treatment modality includes wide resection with ipsilateral radical orchidectomy [15, 16]. The use of adjuvant therapy (radiation and chemotherapy) is controversial [16]. However, adjuvant radiation therapy may be required for positive resection margins. It reduces local recurrence but has no effect of the survival rate [16]. A prolonged follow-up is required since local recurrence occurs several years after treatment [16]. In our case, surgical excision alone was adequate and is consistent with literature [3, 4].

## CONCLUSION

Primary scrotal lipomas are rare but should be considered in the differential diagnosis of unusual scrotal masses. USG is a useful diagnostic tool for scrotal pathologies in resource-limited settings.

## CONSENT

Written informed consent was taken from the patient for publication of this report.

## CONFLICT OF INTEREST

None declared.
